# Clinical Characteristics of Hospitalized COVID-19 Patients Who Have Gastrointestinal Bleeds Requiring Intervention: A Case-Control Study

**DOI:** 10.7759/cureus.26538

**Published:** 2022-07-03

**Authors:** Ahmad Abulawi, Ali Al-Tarbsheh, Annie Leamon, Paul Feustel, Amit Chopra, Asra Batool

**Affiliations:** 1 Internal Medicine, Albany Medical Center, Albany, USA; 2 Albany Medical College, Albany Medical Center, Albany, USA; 3 Neuroscience and Experimental Therapeutics, Albany Medical College, Albany, USA; 4 Pulmonary and Critical Care Medicine, Albany Medical Center, Albany, USA; 5 Gastroenterology, Albany Medical Center, Albany, USA

**Keywords:** gastrointestinal bleed, esophagogastroduodenoscopy, endoscopy, gi bleed, critical care, covid 19, colonoscopy

## Abstract

Background

Coronavirus disease 2019 (COVID-19) is widely recognized as a disease that affects the respiratory system, although it can also present with significant extrapulmonary symptoms. Very few studies have suggested an increased risk of gastrointestinal (GI) bleeding. This study aimed to elucidate the incidence, etiology, risk factors, and outcomes of clinically significant GI bleeding requiring endoscopic intervention in patients with COVID-19.

Methods

This is a case-control (1:2) retrospective analysis of all hospitalized adult patients with COVID-19 infection admitted between March 1, 2020, and January 5, 2021, in which we compared patients with upper and lower GI bleeds to those without. Cases are defined as patients hospitalized with COVID-19 who had a GI bleed requiring intervention while controls are defined as patients hospitalized with COVID-19 who did not have a GI bleed. Of 1002 patients admitted to the Albany Medical Center with COVID-19 infection, there were 76 confirmed cases of GI bleeding. These patients were compared to a control group composed of randomly selected patients with COVID-19 infection who were admitted to Albany Medical Center over the same time period. We assessed patients for in-hospital mortality, ventilator-free days on day 28, ICU-free days on day 28, and hospital-free days on day 28. Additional information collected included demographic information, comorbid conditions, COVID-19 treatments received, endoscopy findings, endoscopic treatment received, and if the patients required a packed red blood cell transfusion.

Results

Out of 1007 patients hospitalized with COVID-19, 76 (8%) had a GI bleed requiring endoscopic intervention. Peptic ulcer disease in the stomach or duodenum was the most common finding. The use of steroids, antiplatelet agents, and anticoagulation was not associated with an increased risk of GI bleed in COVID-19 patients. The GI bleed group required ICU care in 37% (28/76) compared with 21% (32/152) in the control group, which was statistically significant (p=0.012; chi-square test). Length of hospital stay was longer in the GI bleed group (median 16 days IQR: 8 to 29 versus 7 days, IQR:4 to 16; p<0.001, Mann Whitney test).

Conclusion

Length of hospital stay and ICU level of care was higher in the GI bleed group of patients with COVID-19. ICU level of care was noted to be associated with an increased risk of GI bleeding. A GI bleed in COVID-19 patients could be from the virus's direct effect on the gut mucosa or stress-induced bleeding like any other severely sick ICU patient; however, this needs to be explored in future studies.

## Introduction

Severe acute respiratory syndrome coronavirus 2 (SARS-COV-2) is most commonly associated with respiratory illness [1]. However, multiple studies have shown that SARS-COV-2 is associated with extrapulmonary complications, including in the gastrointestinal system. Importantly, recent studies have shown that patients hospitalized with coronavirus disease 2019 (COVID-19) are at risk of a gastrointestinal (GI) bleed [2-4]. The reported incidence of GI bleeds was 2-3% in hospitalized patients with COVID-19 and was noted to be higher in ICU patients [3,5-6]. The widespread use of anticoagulation and corticosteroids in these patients could lead to a significant GI bleed [7-9]. However, there is a significant lack of data in assessing risk factors and outcomes of hospitalized patients with COVID-19 infection who developed a GI bleed. This retrospective analysis aimed to elucidate incidence, etiology, risk factors, and outcomes of clinically significant upper and a lower GI bleed with COVID-19 infection requiring endoscopic intervention.

## Materials and methods

This is a case-control (1:2) retrospective analysis of all hospitalized adult patients with COVID-19 infection who were admitted to Albany Medical Center between the dates of March 1, 2020, and January 5, 2021. This study was approved by the Albany Medical Center institutional review board and registered under protocol number 5825. Given the retrospective nature of our study, requirements for informed written consent were waived. COVID-19 was diagnosed by reverse transcription-polymerase chain reaction (RT-PCR) from a nasopharyngeal swab. GI bleeding was defined as overt upper and/or lower GI bleeding, including melena, hematochezia, hematemesis, or coffee-ground emesis, with a significant drop in hemoglobin of 2 g/dL or more from their baseline on admission with or without hemodynamic instability that required endoscopic intervention (i.e. upper endoscopy and/or colonoscopy).

Inclusion criteria included adult patients 18 years of age and above who were admitted with COVID-19 and a clinically significant GI bleed as described above. The following COVID-19 patients were excluded: a) pregnant; b) incarcerated; c) patients with a GI bleed who did not receive endoscopic interventions.

A total of 1007 patients admitted with a COVID-19 diagnosis during the study period were manually reviewed in our electronic medical record for a GI bleed according to our definition. In addition to GI consultation notes, an endoscopy database (ProVation, Minneapolis, Minnesota) was used to identify all patients during the study period who underwent endoscopy. After analyzing all data, 76 confirmed cases with a GI bleed requiring endoscopic intervention were identified. Our control group was selected randomly from the cohort of COVID-19-positive patients without a GI bleed in a 1:2 ratio using a random number generator.

Patient data collected included: a) Demographics; b) Comorbidities; c) Laboratory parameters; d) COVID-19 treatment received: Remdesivir, anti-coagulation, corticosteroids; e) endoscopic findings; f) intervention done; clips, cautery, epinephrine injection or rectal packing; g) packed red blood cells transfusion; and h) outcome measured at day 28, including ventilator-free days, ICU-free days, and hospital-free days; and overall in-hospital mortality.

We performed analyses to determine the incidence of gastrointestinal bleeds in hospitalized patients with COVID-19 infection. We also compared the clinical characteristics and outcomes of patients with COVID-19 infection who developed a significant gastrointestinal bleed requiring endoscopic intervention and the in-hospital mortality, ventilator-free days on day 28, ICU-free days on day 28, and hospital-free days on day 28 to the control group.

Statistical analysis

Continuous variables were represented by mean and standard deviation for normally distributed variables (age, BMI, and Hgb) and by the median and interquartile range (25th to 75th percentile) for non-normally distributed variables. Statistical inference for continuous variables was by the t-test for normally distributed variables and otherwise by the Mann-Whitney non-parametric test. Significance accepted at p<0.05. Categorical data were presented as frequencies and percentages with inference by Pearson’s chi-square test or Fisher’s exact test if the expected value in any cell was less than five.

**Figure 1:**
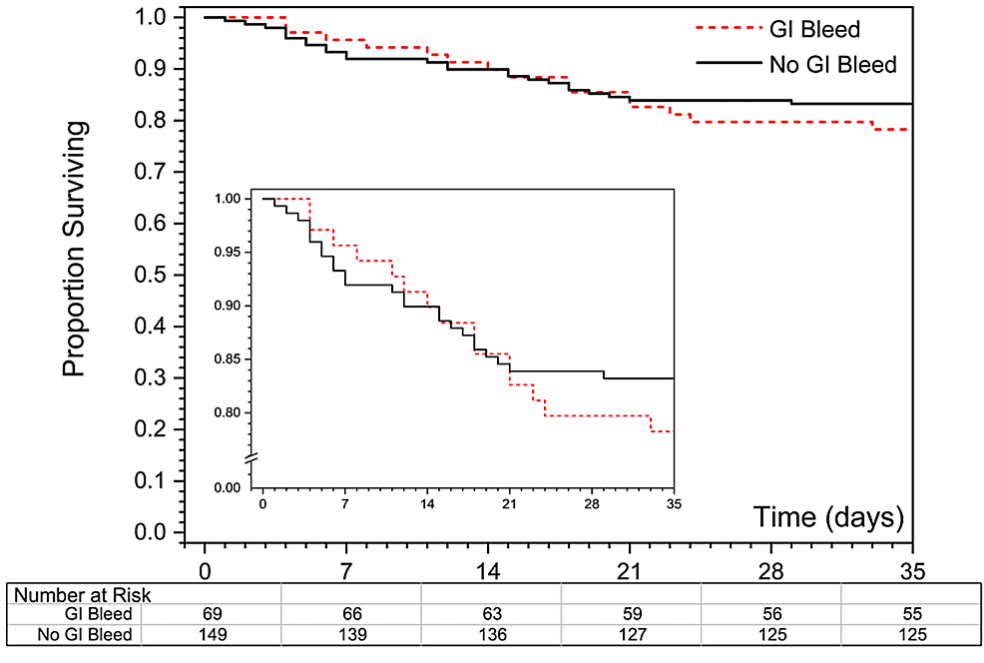
Panel A shows Kaplan-Meier curves of proportion surviving in the two groups. Insert shows survival on an expanded scale. Patients discharged from the hospital were considered to have survived at least 35 days. Curves are not statistically significantly different (p=0.44 by the log-rank test). Panel B shows a specific number of patients with and without a GI bleed on days 0, 7, 14, 21, 28, and 35, respectively.

Kaplan-Meier survival curves were plotted to compare the in-hospital survival of COVID-19 patients with and without a GI bleed. The log-rank test was used to assess the difference between survival curves and the chi-square test was used to calculate the difference in survival between two groups at 120 days. Analysis was performed using Minitab (v.19.2020.1; Minitab, LLC) and R (v.3.6.1) statistical software (R Core Team (2020). R: A language and environment for statistical computing. R Foundation for Statistical Computing, Vienna, Austria) (Figure 1).

## Results

A total of 76 hospitalized patients with COVID-19 were identified as having a clinically significant GI bleed requiring endoscopic intervention. The overall incidence of a clinically significant GI bleed was 76/1007=8%. The cohort was comprised predominantly of the male gender (41/76, 54%) with a mean age of 67 (SD=16) years. The most common comorbidities noted were 54% (41/76) hypertension, followed by 33% (25/76) diabetes mellitus.

A GI bleed was diagnosed at median day 9 (IQR: 6-12) after hospitalization. In all cases, 100% of the patients (76/76) underwent esophagogastroduodenoscopy and 37% (28/76) underwent lower GI endoscopy in the form of either colonoscopy or flexible sigmoidoscopy. On upper endoscopy, peptic ulcer disease in the stomach or the duodenum was the most common finding in 47% (36/76), followed by gastritis and erosions in 9% (7/76). Variceal bleeding from gastroesophageal varices was noted only in one patient. On lower endoscopy, rectal ulcers were the most common finding in 53% (12/28). Endoscopic treatment was performed in 23 patients, with the use of endoclips being the most common intervention. Two patients required angiography and embolization, and four patients required rectal packing for bleeding rectal ulcers. Hemostasis was achieved in all cases, 84% (64/76) of the patients required an average of three units of packed red blood cell transfusion. The endoscopic findings and treatments are described in Table 1.

**Table 1:** Endoscopic Finding and Management Of COVID-19 Patient with Upper GI Bleeding

Variables	Patients With Upper GI Bleed N=76	Patients with Lower GI Bleed N=28
Endoscopic Findings -N (%)		
Normal	30 (39%)	9 (32%)
Esophagitis	2 (3%)	N/A
Gastritis/Erosion	7 (9%)	N/A
Gastroduodenal ulcer	36 (47%)	N/A
Esophageal Varices	1 (1%)	N/A
Rectal Ulcer	N/A	15 (53%)
Diverticular Bleed	N/A	2 (7%)
Internal Hemorrhoid	N/A	1 (3.5%)
Colitis	N/A	1 (3.5%)
Ulcer, Forrest Classification - N (%)		
Ia	15 (40%)	5 (33%)
Ib	12 (32%)	5 (33%)
IIa	6 (16%)	3 (20%)
III	4 (10%)	2 (13%)
Interventions - N (%)		
Epinephrine Injection	2 (5%)	N/A
Cautery	3 (8%)	N/A
Endoclip	6 (16%)	5 (33%)
Variceal Banding	1 (3%)	N/A
Embolization	1 (3%)	1 (7%)
Rectal Packing	N/A	4 (27%)

We randomly selected 152 controls as mentioned in the methods. The baseline characteristics were similar in the cases and controls as listed in Table 2. The average Hgb was lower in the GI bleed group compared with the controls and this was statistically significant. As expected, packed RBC transfusions were higher in the GI bleed group compared to the control group (84% vs 15%). Higher values of white blood cell count and C-reactive protein were observed in the GI bleed group, but these were not statistically significant. There was no statistical difference between the two groups in steroids, aspirin, or NSAID usage as demonstrated in Table 2. Anti-coagulation usage was almost four-fold higher in the GI bleed group but was not associated with an increased risk of GI bleed.

**Table 2:** Characteristics of the Cohort Population * p-values for comparison of cases vs randomly selected controls by the t-test for normally distributed variables (age, BMI, and hemoglobin) and the chi-square test for categorical variables ** p-value by the Mann-Whitney test *** p-value by Fisher’s exact test SD - standard deviation; BMI - body mass index; CRP - C-reactive protien; BUN - blood urea nitrogen; NSAIDs - non-steroidal anti-inflammatory drugs; MV - mechanical ventilation

Variables	All Patients N=1007	Cases N=76	Controls N=152	P-Value* (Case vs Control)
Age-Year; Mean ± SD	63 ± 18	67 ± 16)	65 ± 16	0.29
Sex- N (%)				0.39
Male	565 (56%)	41 (54%)	91 (60%)	
Female	442 (44%)	35 (46%)	61 (40%)	
Ethnicities- N (%)				0.31
White	599 (59%)	46 (61%)	103 (68%)	
Black	202 (20%)	13 (19%)	30 (20%)	
Hispanic	37 (4%)	3 (4%)	4 (2.6%)	
Asian	57 (6%)	3 (4%)	6 (4%)	
Unknown/Unreported	112 (11%)	11 (14%)	9 (6%)	
BMI- Kg/m^2^; Mean ± SD	29.9 ± 8.3	29.4 ± 8.7	29.7 ± 7.6	0.83
Comorbidities - N (%)				
Diabetes Mellitus	290 (29%)	25 (33%)	48 (31%)	0.84
Coronary Artery Disease	180 (18%)	14 (18%)	29 (19%)	0.91
End-Stage Renal Disease	33 (3%)	1 (1%)	8 (5%)	0.28***
Current or Ex-Smoker	246 (24%)	20 (26%)	27 (18%)	0.14
Chronic Anemia	136 (14%)	40 (53%)	17 (11%)	<0.001
Laboratory Values on Admission; Mean±SD or Median (Interquartile Range)				
Hemoglobin at Presentation	12.6 ± 3.1	10.1 ± 2.2	12.6 ± 2.4	<0.001
Ferritin (ng/mL)	367 (146 - 816)	393 (177 - 1016)	356 (154 - 731)	0.23**
CRP (mg/L)	74 (27 – 143)	171 (90 - 432)	67 (31 - 129)	0.14**
D-Dimer (mg/L)	1.06 (0.58 – 2.04)	2.10 (1.17 – 10.16)	1.10 (0.69 – 2.09)	0.003**
White Blood Cells (K/ulu)	6.90 (4.80 – 9.60)	7.80 (4.30-10.85)	6.50 (4.80-8.55)	0.27**
Highest BUN	31 (18 -59)	40 (20 - 61)	28 (18 - 71)	0.58**
Treatment - N (%)				
Corticosteroid	468 (46%)	31 (41%)	64 (42.1%)	0.85
Aspirin or NSAIDs	150 (15%)	7 (10%)	25 (16%)	0.12
Therapeutic Anti-Coagulants	96 (9.5%)	16 (21%)	9 (6%)	0.001
Red Blood Cell Transfusion	205 (20%)	64 (84%)	10 (15%)	<0.001
Required MV	128 (13%)	18 (24%)	14 (9%)	0.004
Days of MV in Those With MV; Median (IQR)	5 (2 - 11)	11.5 (4.5 – 15.3)	5.5 (1.7 – 12.7)	0.21**
Outcome				
Mortality; N (%)	163 (16.2%)	15 (20%)	25 (16%)	0.66
28 Hospital Free Days; Median (IQR)	19 (0 - 24)	5 (0 - 19)	19 (0 - 23)	<0.001**
Required ICU care; N (%)	270 (27%)	28 (37%)	32 (21%)	0.02
28 ICU Free Days in those requiring ICU care; Median (IQR)	10 (0 - 23)	10 (0 – 22)	5.5 (0 - 22)	0.83

The GI bleed group required ICU care in 37% (28/76) compared with 21% (32/152) in the control group, which was statistically significant (p=0.012; chi-square test). Length of hospital stay was longer in the GI bleed group (median 16 days IQR: 8 to 29 versus 7 days, IQR:4 to 16; p<0.001, Mann-Whitney test). The 28-day all-cause mortality was not statistically significant between the two groups (21.7% in the GI bleed group vs. 16.8% in the non-GI bleed; P =0.38).

## Discussion

In our analysis, 76 patients hospitalized with COVID-19 infection developed a GI bleed requiring endoscopic intervention. Our study found the incidence of GI bleeds in these patients was noted to be 8% as compared to previous studies showing a lower incidence of 1.5%-5.5% in the hospitalized patients in critical care units without COVID-19 infection [7,10]. All patients (76) with a GI bleed received EGD with the most common finding being peptic ulcer disease (47%, 36/76). Interestingly, anticoagulation and NSAID use do not seem to increase the risk of GI bleed in this patient population. Also, no statistically significant difference was noted in overall mortality in COVID-19 patients with or without a GI bleed. More patients were noted to be in the ICU setting among the GI bleed cohort, which could be one of the risk factors predisposing them to have significant GI bleed.

Limited literature has examined the incidence of GI bleeds in hospitalized patients with COVID-19 infection. The estimated incidence has been reported from 0.5% to 3%. Mauro et al. described the incidence of upper GI bleeds in the COVID-19 population in their multicentric study. The reported incidence of GI bleeds in their study was 0.47%, which was even lower than the reported rate of GI bleeds in non-COVID-19 patients. However, the generalizability of their findings is limited given that they excluded critically ill patients and patients with a lower GI bleed [5]. In the larger multicentric study from New York City, Trindade et al. reported an incidence of 3%. In their cohort population, about one-third had evidence of GI bleed on presentation; this may be an overestimation of the true association between COVID-19 and GI bleed. Also, they could not differentiate if the anticoagulation dose was therapeutic or prophylactic due to dose changes and switching between prophylactic and therapeutic doses for any given patient [3]. Our study reported the incidence of 8% of GI bleeding requiring endoscopic intervention, which is substantially higher than the previously reported studies. This difference may be due to patient selection, population size, and the difference in criteria defining a GI bleed. In our study, we only included patients with GI bleeds who required endoscopic intervention with clear inclusion criteria and definition of GI bleed.

The incidence of GI bleeds in non-COVID critically ill patients has been reported as anywhere from 1.5% to 5.5%, which is nearly similar to patients with COVID infection [11]. The notable risk factors for the increased risk of bleed were the duration of mechanical ventilation lasting more than 48 hours, use of prophylactic anticoagulation, and stress-induced ulceration [7,10]. Our study produced similar results to those of Cook et al., finding that respiratory failure, as well as physiologic sources of stress such as sepsis and hypotension, are commonly associated with ulcers in critically ill patients [10].

There are limited studies examining the risk factor for a GI bleed in hospitalized COVID-19 patients. The majority of these studies have shown no association between GI bleeds and the use of anticoagulation or steroids in this population, which aligns with our findings. However, one single-center study from Bronx Lebanon Hospital showed an increased risk of GI bleeds in COVID-19 patients with the use of steroids and anticoagulation. There are several limitations to their study, including selection bias, sample size, and definition for GI bleed [2]. Few studies also looked at the effect of GI bleeding on the outcomes in this specific population such as length of hospital stay, complications, mortality, etc. The consensus from these studies has not shown any impact of GI bleeding on the overall outcomes [7,12-13]. Our study showed similar results, as there was no difference in the clinical outcomes between the GI bleed and control groups.

There are several potential mechanisms of GI bleeds in hospitalized patients with COVID-19, which include the development of an inflammation-induced coagulopathy, direct damage of the virus on the gastrointestinal mucosa, stress ulcer from critical illness, and other factors, such as thromboprophylaxis, and steroids, which represent an additional risk factor for bleeding. At this point, it is not clear which is the dominant mechanism for the increased incidence of GI bleeds in COVID-19 patients. Marasco G et al. believe it may be because of critical illness and stress-induced ulcers, as reflected by a higher percentage of GI bleeding occurring in patients who are hospitalized with critical status in the ICU [14]. In our study, most of the bleeding happened after a few days of hospitalization, which suggests that bleeding is more likely due to factors related to critical illness or treatment-related rather than primary viral-induced mucosal injury.

To our knowledge, this is the first study aimed to study significant GI bleed in hospitalized patients with SARS-COV-2 infection requiring endoscopic intervention. Our study has several strengths, including a clear definition of GI bleeding in patients hospitalized with COVID-19 that required endoscopic intervention, a case-control study with a detailed comparison of clinical characteristics between the study group and the control group, and a relatively large number of the patient population who underwent endoscopic evaluation than previously reported. We also acknowledge some limitations in the study, including it being a retrospective, single-center study, a possible selection bias without randomization, and a lack of generalization to assess the risk of GI bleed in all hospitalized patients with COVID infection.

## Conclusions

In summary, we found that 8% of critically ill patients with COVID-19 infection developed a GI bleed requiring endoscopic intervention. Patients with longer ICU stays were likely to have a higher risk of GI bleed. Anticoagulation was not associated with an increased risk of a significant GI bleed in our study group. There was no statistically significant difference in the 28-day all-cause mortality between the two groups. Further large prospective studies are required to assess the risk and the pathophysiology of GI bleeding in hospitalized patients with COVID-19.
